# Recent Developments in Polymer Inclusion Membranes: Advances in Selectivity, Structural Integrity, Environmental Applications and Sustainable Fabrication

**DOI:** 10.3390/membranes15080249

**Published:** 2025-08-19

**Authors:** Anna Nowik-Zając, Vira Sabadash

**Affiliations:** 1 Faculty of Science and Technology, Jan Dlugosz University in Czestochowa, Armii Krajowej 13/15, PL 42200 Czestochowa, Poland; 2 Department of Ecology and Sustainable Environmental Management, Lviv Polytechnic National University, UA 79000 Lviv, Ukraine; vira.v.sabadash@lpnu.ua

**Keywords:** polymer inclusion membranes, carrier-mediated transport, green membrane technologies, deep eutectic solvents, ionic liquids, heavy metal removal, gas separation, membrane stability, analytical sensing membranes, sustainable separation processes

## Abstract

Polymer inclusion membranes (PIMs) have undergone substantial advancements in their selectivity and efficiency, driven by their increasing deployment in separation processes, environmental remediation, and sensing applications. This review presents recent progress in the development of PIMs, focusing on strategies to enhance ion and molecule selectivity through the incorporation of novel carriers, including ionic liquids and task-specific extractants, as well as through polymer functionalization techniques. Improvements in mechanical and chemical stability, achieved via the utilization of high-performance polymers such as polyvinylidene fluoride (PVDF) and polyether ether ketone (PEEK), as well as cross-linking approaches, are critically analyzed. The expanded application of PIMs in the removal of heavy metals, organic micropollutants, and gas separation, particularly for carbon dioxide capture, is discussed with an emphasis on efficiency and operational robustness. The integration of PIMs with electrochemical and optical transduction platforms for sensor development is also reviewed, highlighting enhancements in sensitivity, selectivity, and response time. Furthermore, emerging trends towards the fabrication of sustainable PIMs using biodegradable polymers and green solvents are evaluated. Advances in scalable manufacturing techniques, including phase inversion and electrospinning, are addressed, outlining pathways for the industrial translation of PIM technologies. The review concludes by identifying current limitations and proposing future research directions necessary to fully exploit the potential of PIMs in industrial and environmental sectors.

## 1. Introduction

### 1.1. Overview of Polymer Inclusion Membranes (PIMs)

Polymer inclusion membranes (PIMs) are a unique type of liquid membrane system. They involve the trapping of a selective carrier and often a plasticizer within a solid polymer matrix. This setup creates a hybrid structure that combines the high selectivity and transport methods typical of supported liquid membranes (SLMs) with the strength and versatility of polymer films. Since they were first developed in the early 1990s, PIMs have become useful materials for various separation and sensing tasks, especially in water treatment, hydrometallurgy, gas purification, and analytical chemistry.

**Figure 1:**
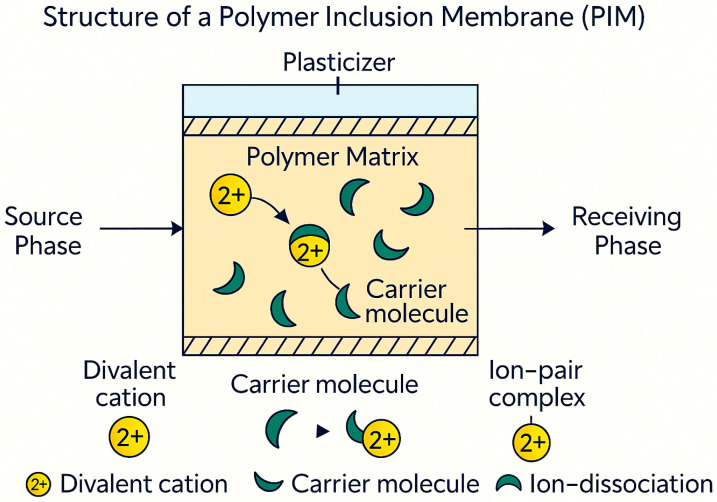
Schematic diagram of the structure of PIMs.

In a typical PIM, the active transport agent (carrier) is evenly spread throughout a polymer matrix. This matrix may include substances such as cellulose triacetate (CTA), polyvinyl chloride (PVC), or polyvinylidene fluoride (PVDF). A plasticizer is sometimes added to improve flexibility and diffusion. The carrier interacts selectively and reversibly with the target species at the membrane surface. This interaction allows transport through cycles of complexation and decomplexation. This carrier-mediated transport method allows for precise adjustments in selectivity by changing the carrier chemistry, the membrane’s composition, and interactions between polymer and carrier. The structure of a PIM is illustrated in Figure 1.

While polymer inclusion membranes have emerged as a promising class of selective separation materials, it is essential to evaluate their performance in comparison with conventional membrane technologies such as reverse osmosis (RO), nanofiltration (NF), and ceramic membranes. These mature techniques have long dominated the industrial separation landscape due to their high throughput, mechanical robustness, and scalability. Reverse osmosis and nanofiltration are pressure-driven processes commonly used in water purification and desalination. While effective for the bulk separation of ions and molecules, they often face challenges such as high energy consumption, fouling, and limited selectivity for specific target species. RO membranes exhibit nearly complete ion rejection but lack the tunable specificity typical of PIMs. Additionally, their operation usually requires high pressures of up to 60 bar, which can be a limiting factor in resource-constrained environments. PIMs function at ambient pressure. They can be customized for highly selective separations by using specific carriers designed for targeted ions. For example, Aliquat 336 is effective for separating chromium (VI), while D2EHPA is used for rare-earth elements [1,2]. This selectivity enables the efficient extraction of trace contaminants or valuable metals from complex matrices where RO and NF would indiscriminately remove both desired and interfering species. For instance, Uğur et al. [3] demonstrated the selective separation of Zn^2^^+^ and Cd^2^^+^ using a PIM that contains calix[4]resorcinarene. They achieved separation factors that cannot be obtained with conventional membranes. Furthermore, PIMs are less prone to biofouling due to their dense structures and hydrophobic nature. Their regeneration can be accomplished simply through pH manipulation instead of backflushing or chemical cleaning, thereby reducing operational costs and complexity. While PIMs generally exhibit lower flux than RO or NF membranes, their advantages in selectivity and ease of operation make them particularly well suited for analytical applications, resource recovery, and selective remediation [4]. Ceramic membranes are well known for their outstanding resistance to heat and chemicals, making them highly effective in challenging environments. They are commonly used in applications such as hot gas filtration, chemical wastewater treatment, and food processing [5]. These membranes can function at temperatures exceeding 300 °C and can withstand highly acidic or basic conditions, surpassing the durability of many polymeric materials. However, they exhibit low flexibility and high brittleness and often require significant capital investment for fabrication and operation due to the sintering process and use of metal oxide precursors (e.g., alumina and zirconia). PIMs generally exhibit lower stability under extreme conditions; however, they provide high selectivity for target analytes due to the inclusion of task-specific extractants and functional carriers. For example, Aliquat 336-based PIMs have demonstrated excellent selectivity for Cr(VI) and As(V) from real wastewater samples under ambient conditions, a property that ceramic membranes cannot exhibit without pre-treatment or functionalization steps [6,7,8]. Moreover, ceramic membranes typically perform size-based (sieving) separations (micro-, ultra-, and nanofiltration). At the same time, PIMs operate through facilitated transport mechanisms, allowing for the separation of ions with similar sizes and charges—a significant advantage in rare-earth element recovery and analytical preconcentration [9]. Ceramic membranes are not naturally compatible with hydrophobic carriers, such as calixarenes or organophosphorus compounds, because of their inorganic surface chemistry. In contrast, polymer inclusion membranes (PIMs) allow for the uniform dispersion of these extractants within the polymer matrix. This capability enables the recognition and transport of specific ions [10,11,12,13].

Polymer inclusion membranes offer several advantages over traditional separation methods [14,15]. They facilitate transport through carrier-mediated diffusion and can operate under ambient pressure and temperature conditions. Additionally, they can be easily customized by chemically modifying the polymer matrix or carrier molecule. PIMs also have low energy requirements and possess intrinsic selectivity, making them well suited for tasks that involve trace-level separation [16]. This includes applications like extracting heavy metals, removing pharmaceuticals, and concentrating analytes for analysis. Furthermore, PIMs can be regenerated simply by changing the phase conditions, eliminating the need for harsh cleaning protocols that are often required in reverse osmosis (RO) or nanofiltration (NF) systems [17]. While PIMs have unique advantages, they also face several challenges, including mechanical strength, long-term stability, and scaling up for industrial use. These issues need to be addressed before PIMs can compete effectively with established membrane technologies. To enhance their performance, hybrid approaches are being explored, such as incorporating nanofillers or utilizing high-performance polymers like PVDF and PEEK [18]. This comparative evaluation emphasizes the unique role of PIMs within the spectrum of membrane technology. Although PIMs are not intended to replace high-throughput systems like reverse osmosis in bulk applications, they provide exceptional selectivity and sustainability for fine separations where traditional membranes are less effective.

Despite their unique advantages, PIMs face several real limitations that must be addressed for wider industrial use. Although there has been remarkable progress in the design and application of PIMs, various practical challenges hinder their large-scale and long-term deployment. One of the main concerns is long-term fouling, particularly when PIMs are used with complex feed matrices such as industrial effluents, wastewater, or biological fluids [19,20]. The accumulation of organic matter, microbial growth, and particulate deposition can block membrane pores, reduce transport efficiency, and ultimately shorten the membrane’s lifespan [21]. While surface modification techniques like PEGylation and fluorination have been explored to reduce fouling, their long-term effectiveness under real-world conditions remains uncertain [21,22,23]. A second critical issue is carrier leaching, particularly in systems where the carrier is physically trapped rather than chemically bonded to the polymer matrix. This problem worsens under dynamic flow conditions or prolonged operation in aggressive media, such as acidic or basic solutions. Carrier leaching not only undermines the selectivity and durability of the membrane, but it also raises environmental concerns due to the potential release of toxic extractants into the treated medium. Operational stability continues to be a significant challenge. Many polymer inclusion membranes perform exceptionally well in controlled laboratory conditions but tend to suffer a decline in performance when exposed to real-world stressors, such as varying temperatures, ionic strengths, pH levels, and continuous pressure. Biodegradable polymer matrices, like polyvinyl alcohol (PVA) or polylactic acid (PLA), are environmentally friendly, but they may experience hydrolytic degradation or a loss of mechanical integrity over long-term use [24]. Additionally, the economic viability of advanced PIM designs is hindered by the high cost of ionic liquids (ILs), particularly task-specific or highly pure types. Although ILs provide enhanced stability, tunable selectivity, and negligible volatility, their synthesis and purification processes are both expensive and energy-intensive. Prices for aprotic ILs commonly used in membrane systems range from approximately USD 2.50 to 50 per kg, significantly higher than those for conventional organic solvents [25]. This financial burden restricts their application in large-volume or low-margin scenarios unless they can be efficiently recovered and reused. Moreover, the incorporation of nanofillers or hybrid components (e.g., MOFs, COFs and carbon nanotubes) also increases the material costs and complexity of fabrication [26]. Addressing these limitations requires a multidisciplinary effort involving material innovation, scalable green fabrication strategies, and the development of standardized testing protocols under realistic operational scenarios. As the field matures, future research must strike a balance between performance, sustainability, and cost to enable the commercial viability of PIM-based systems in diverse separation processes.

In recent years, significant research has focused on enhancing the selectivity, chemical stability, and sustainability of PIMs. A key advancement has been the addition of task-specific ionic liquids (ILs) as carriers. These salts, which are liquid at room temperature, have low volatility, high thermal stability, and adjustable physical and chemical properties. For instance, Hernández-Fernández et al. (2023) showed that using imidazolium-based ILs in PIMs significantly improved the selective separation of heavy metal ions, such as Pb^2+^ and Zn^2+^. This is due to strong coordination between the IL cations and anions [27]. Similarly, in a 2024 review titled “Ionic liquid-based extraction of metal ions via polymer inclusion membranes: a critical review”, Adigun et al. discussed the strong attraction of IL-functionalized PIMs for metal ions and the potential for adjusting selectivity via IL functionalization [28].

In addition to ionic liquids, organophosphorus extractants such as di-(2-ethylhexyl) phosphoric acid (D2EHPA), Aliquat 336, and Cyanex 301 are widely used as carriers. They are effective in transporting metal ions. A recent study by Kazemi and Yaftian (2024) reported the development of a PVDF-HFP-based PIM that includes D2EHPA. This PIM showed excellent selectivity and stability for the extraction of Bi^3+^ ions, maintaining high transport efficiency over multiple reuse cycles [29].

Concurrently, progress in polymer science has enabled the design of PIMs with improved structural integrity and enhanced resistance to degradation. High-performance polymers, such as polyether ether ketone (PEEK), PVDF, and CTA, have shown superior chemical and thermal resistance compared to traditional matrices like PVC. Their use has increased the operational lifespan of PIMs, especially in harsh industrial or environmental conditions. Additionally, polymer cross-linking and blending with hydrophilic additives have strengthened the membranes, decreased brittleness, and reduced carrier leaching.

Beyond performance improvements, recent research has focused on the sustainable production of PIMs. An increasing amount of literature explores the use of biodegradable polymers (e.g., polylactic acid, chitosan), along with green solvents like ethanol, ethyl acetate, and Cyrene™, as ecofriendly alternatives to traditional toxic solvents, such as tetrahydrofuran (THF) and dichloromethane. In a 2025 study titled “Polymer Inclusion Membranes (PIMs) for Metal Separation-Toward Environmentally Friendly Production and Applications”, Senila et al. reviewed the integration of green chemistry principles into PIM production, including solvent replacement, bio-based polymers, and recyclable components [30].

The application scope of PIMs has expanded considerably in recent years. In environmental remediation, PIMs have shown effectiveness in the selective removal of heavy metals (e.g., Cd^2+^, Cu^2+^, Zn^2+^ and Ni^2+^) from industrial wastewater, acid mine drainage, and leachates from electronic waste recycling. Their high separation factors, low energy requirements, and reusability make them attractive options to traditional methods. For example, polymer inclusion membranes based on Aliquat 336 embedded in CTA have shown over 95% removal efficiency for Zn^2+^ from complex aqueous matrices, while maintaining structural stability across ten extraction cycles [31].

Analytical platforms have also been integrated with PIMs to preconcentrate and speciate metal ions, pharmaceuticals, and organic pollutants. The immobilized carrier environment enables the enrichment of trace analytes, improving detection sensitivity in optical and electrochemical sensors. Recent developments include optode-based PIM sensors with colorimetric responses to mercury(II) and silver(I), demonstrating rapid and selective detection in field conditions [32].

The potential of PIMs in gas separation, particularly for CO_2_ capture, is gaining attention. Ionic liquid-modified membranes have shown good results in selectively transporting CO_2_ over N_2_ or CH_4_, offering new possibilities for their application in flue gas treatment and carbon capture technologies. Although gas-selective PIMs are still in the early stages of commercialization, initial studies highlight their scalability and compatibility with low-pressure operations.

Scalable fabrication methods such as phase inversion, solution casting, and electrospinning are currently being explored. These methods aim to shift PIMs from laboratory prototypes to industrial modules. They allow for control over the thickness, porosity, and morphology of the membranes, which affects their transport kinetics and durability. Combining modular units and hybrid membrane systems represents an exciting area in PIM development, particularly for continuous-flow processes.

In summary, polymer inclusion membranes are in a transformative stage marked by the careful design of carriers, sustainable fabrication approaches, and interdisciplinary applications. Their adjustable selectivity, operational stability, and potential for green engineering make them a flexible technology for separation science in the 21st century. The following sections of this review will provide more details on these advancements, focusing on the specific strategies, materials, and applications that are reshaping the scope and potential of PIMs.

### 1.2. Historical Development and Current Research Trends

The concept of polymer inclusion membranes (PIMs) emerged from the broader field of liquid membrane technologies, which began in the mid-20th century. In 1963, Bloch proposed immobilizing liquid extractants in solid polymer supports, enabling selective separations without the phase instability associated with traditional solvent extraction systems [33]. This basic concept set the stage for the development of PIMs. In the 1990s, researchers such as Almeida, Cattrall, and Kolev defined the PIM structure by adding carriers like Aliquat 336 or D2EHPA into polymers such as cellulose triacetate (CTA) or polyvinyl chloride (PVC) [2,9]. They used a carrier-mediated transport mechanism. The analyte would form a reversible complex with the carrier at the source phase, diffuse through the membrane, and decomplex at the receiving phase. This process allowed for the targeted transport of specific species.

During the 2000s, the research focus shifted to mechanistic understanding and optimization. Studies characterized how polymer–carrier ratios, plasticizer concentration, and matrix hydrophobicity influenced membrane permeability, selectivity, and reusability [34]. Researchers also began exploring alternative carriers, such as calixarenes and phosphonic acids, enabling the separation of rare-earth elements and toxic heavy metals under environmentally relevant conditions.

From 2010 onward, PIM applications broadened substantially, particularly into environmental remediation and analytical chemistry. This period saw the emergence of PIMs for removing dyes, pharmaceuticals, and heavy metals from real wastewater matrices. Mechanistic models based on Nernst-Planck equations and Fickian diffusion helped quantify multi-ion systems and predict transport behavior [8]. At the same time, chemically resistant polymers such as PVDF, PEEK, and modified CTA improved durability under harsh chemical conditions.

A breakthrough in PIM technology occurred in the early 2020s: the introduction of ionic liquids (ILs) and deep eutectic solvents (DESs) as task-specific carriers. These carriers offered better chemical adjustment, thermal stability, and selectivity for both inorganic and organic targets. In a detailed 2024 review, Adigun et al. noted that IL-based PIMs are superior in terms of extraction capacity, chemical resistance, and long-term reusability, especially when handling complex effluents [28]. A similar wave of innovation focused on using DES-based carriers, leveraging their biodegradability and lower toxicity. Simultaneously, sustainability in PIM fabrication became a central research priority. A 2025 review by Senila et al. summarized advances in green PIM production, including the use of biodegradable polymers (e.g., PLA and chitosan), biofillers (e.g., acetylated lignin), and green solvents (e.g., Cyrene™, 2-MeTHF, and ethyl acetate) [30]. Studies have demonstrated that these materials could maintain high transport efficiencies while reducing the environmental footprint of membrane production. For instance, Kaczorowska et al. (2022) fabricated CTA-based PIMs incorporating lignin for Ni(II) recovery with industrial wastewater relevance [35].

At the same time, structural and functional improvements to PIMs gained traction. Kazemi and Yaftian (2024) developed PVDF-HFP membranes incorporating D2EHPA, which exhibited a transport efficiency of >90% Bi(III) and strong operational stability over multiple reuse cycles [29]. Other innovations included cross-linked systems, nanocomposites, and biofiller-enhanced matrices that combined performance with material resilience [36]. A particularly active research trend is the integration of PIMs with sensing technologies. Colorimetric and electrochemical PIM-based sensors have been successfully developed for detecting mercury(II), silver(I), and phosphate ions, showing fast response times and high sensitivity [35,37,38]. These sensors are being adapted for field-deployable monitoring systems with environmental and biomedical applications.

**Figure 2:**
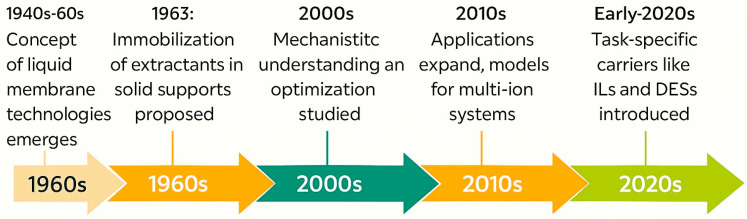
Historical Timeline of PIM Development.

In parallel, advances in scalable fabrication techniques such as phase inversion, electrospinning, and solvent casting enable industrial translation. These methods enable precise control over membrane thickness, porosity, and mechanical strength, which are crucial for continuous-flow operation and long-term use in real-world systems. Figure 2 shows a historical timeline of PIM development, giving a visual overview of this progression. Table 1 provides a detailed breakdown of important periods, innovations, and representative publications.

This development shows that PIMs have changed from basic solvent-immobilization systems to customizable, sustainable, and increasingly scalable materials. The combination of green chemistry, new materials, and process engineering ensures that PIMs will remain important in separation science and sustainable technologies in the future.

## 2. Advances in the Selectivity and Functionalization of PIMs

Selectivity is the defining characteristic of polymer inclusion membranes (PIMs), distinguishing them from other separation technologies. Selectivity can be dramatically enhanced by two main strategies: (1) designing advanced carriers such as ionic liquids (ILs), deep eutectic solvents (DESs), and task-specific extractants, and (2) modifying the chemical and physical structure of the polymer matrix. These complementary methods provide synergistic control over transport behavior, enabling PIMs to selectively extract target species from increasingly complex mixtures, such as industrial leachates, environmental waters, and biological fluids.

### 2.1. Novel Carriers and Extractants: Ionic Liquids, Task-Specific Molecules

The transport carrier is essential to a polymer inclusion membrane (PIM). It plays a direct role in recognizing, complexing, and moving target species. In recent years, there has been significant progress in this field. This is especially true for the use of ionic liquids (ILs), deep eutectic solvents (DESs), and task-specific organic extractants. These offer customizable chemical environments that dramatically enhance selectivity.

Ionic liquids are room-temperature molten salts composed of organic cations—such as imidazolium, pyridinium, or phosphonium—and large, non-coordinating anions like [PF_6_]^−^ or [NTf_2_]^−^. Their structural diversity and negligible volatility make them attractive alternatives to traditional liquid extractants. When embedded in PIMs, ILs can provide tailored ion–pair interactions and controlled diffusion environments. For example, membranes formulated with 1-octyl-3-methylimidazolium bis(trifluoromethanesulfonyl)imide ([C_8_mim][NTf_2_]) and Cyanex 301 in a PVDF-HFP matrix demonstrated high selectivity for Co^2+^ and Ni^2+^, enabling over 90% recovery of Co^2+^ from lithium-ion battery leachate. The separation factor (α) for Co^2+^ over Li^+^ exceeded 30, making this system highly relevant for the recovery of critical metals from electronic waste [27].

In addition to imidazolium-based ILs, phosphonium ionic liquids, such as trihexyl(tetradecyl)phosphonium chloride ([P_66614_][Cl]), have emerged as powerful carriers for soft acid metals like Ag^+^ and Hg^2+^. Their large hydrophobic cations provide a highly non-polar environment that facilitates selective complexation through soft—soft Lewis interactions. Importantly, phosphonium ILs exhibit high thermal and chemical stability, making them suitable for challenging aqueous matrices and industrial effluents [28].

A growing class of carrier media is deep eutectic solvents (DESs). These are formed by the eutectic mixture of a hydrogen bond donor and acceptor (e.g., choline chloride with glycerol, urea, or ethylene glycol). DESs have many useful properties similar to ILs but are usually easier to synthesize, biodegradable, and more cost-effective. DES-based PIMs have shown potential in selectively separating of Cd^2+^, Zn^2+^, and Pb^2+^, performing comparably to IL-based systems. These systems are particularly beneficial in low-resource settings or when the sustainability of membranes is important [47]. DESs can also enhance membrane antifouling properties, consistent with trends described in Kandel et al. (2025) in natural antifouling agents in membrane systems [48]. The use of these solvents not only facilitates selective ion separation but also reduces biofouling through various mechanisms, including the modification of hydrophilic surfaces, disruption of bacterial adhesion, and generation of osmotic stress in microbial cells. In addition, DESs composed of naturally derived constituents such as betaine, glycerol, or lactic acid exhibit antimicrobial activity, further extending their potential role in sustainable membrane engineering. This dual functionality makes DES-based carriers highly promising for developing multifunctional PIMs suitable for complex wastewater matrices.

Another group of extractants that has attracted significant interest includes task-specific molecules, such as calixarenes, crown ethers, and phosphorylated ligands. These molecules provide size-selective and highly directional complexation mechanisms, resembling processes of biological recognition. For example, calix[4]arene derivatives bearing phosphine oxide groups have been incorporated into CTA-based membranes for the separation of rare-earth elements, achieving separation factors > 100 for La^3+^ over Eu^3+^ [49,50]. Their rigid cavity structure and ability to form stable inclusion complexes make them ideal for high-precision separation. On the other hand, crown ethers are effective for alkali metal cations, particularly in PIMs where ion size and hydration shell properties matter. A membrane containing 18-crown-6 and Aliquat 336 showed high selectivity between Na^+^ and K^+^. This provides precise control in systems such as nutrient recovery or water softening [50]. Importantly, greener carriers such as DESs, task-specific ILs, and bio-based extractants are not merely auxiliary components but are equally vital as membrane-support materials for achieving comprehensive sustainability. While polymer matrices dictate the mechanical properties and stability of PIMs, it is the carrier or extractant that governs transport selectivity, efficiency, and reusability. A poorly selected extractant, even when combined with a green polymer, may jeopardize environmental safety due to its toxicity, potential for bioaccumulation, or leaching. Thus, carriers must be optimized for both their functionality and ecological compatibility.

The effectiveness of these carriers depends not only on their chemical attraction but also on their compatibility with the polymer matrix, their diffusion coefficients, and their tendency to leach into nearby phases. Carriers with high molecular weights or lipophilicity tend to remain immobilized within the membrane, maintaining long-term performance. Meanwhile, functional groups capable of hydrogen bonding or ion pairing ensure the reversibility of complexation, which is essential for facilitated transport. Natural or semi-synthetic carriers derived from renewable resources present promising benefits as safer alternatives. For instance, cyclodextrins can selectively encapsulate hydrophobic organic pollutants through host–guest interactions. In the context of PIMs, CDs are being studied as bio-based carriers or functional modifiers that enhance the selective extraction of target analytes through host–guest interactions and surface adsorption mechanisms. For example, β-cyclodextrin-based membranes have been successfully utilized for removing endocrine-disrupting compounds (e.g., bisphenol A), phenolic pollutants, and certain pharmaceuticals from aqueous solutions [51]. Chemical modifications can be applied to CDs (cyclic compounds) to enhance their properties. For example, they can be carboxymethylated or amino-functionalized to introduce donor atoms that can coordinate with metal ions such as Pb^2^^+^, Cd^2^^+^, and Cu^2^^+^. This coordination occurs through ion–dipole or hydrogen bonding interactions [52,53,54,55]. These modifications have been shown to improve both the selectivity and stability of the membrane phase. Cyclodextrins are highly versatile and adhere to the principles of green chemistry. They are biodegradable, renewable (as they are produced from the enzymatic degradation of starch), and have low environmental toxicity. Integrating cyclodextrins into polymer inclusion membranes (PIMs) has been shown to minimize leaching and enhance the recyclability of the membranes [56]. Moreover, cyclodextrins are highly compatible with various eco-friendly polymer matrices, such as chitosan, poly(lactic acid), and poly(vinyl alcohol). This compatibility not only improves the environmental profile of the membranes but also strengthens their structural integrity [57]. Additionally, modified tannins or polyphenols are capable of chelating heavy metals. In the study by Nowik-Zając et al., calix[4]pyrrole derivatives showed exceptional selectivity for Ag^+^ ions through anion coordination mechanisms, demonstrating fast transport kinetics and minimal leaching [58]. Similarly, calixresorcinarene-based carriers achieved over 90% removal of Pb^2^^+^, exhibiting high reusability and aligning with both performance and green chemistry objectives [58,59]. The effectiveness of task-specific ionic liquids (ILs) and deep eutectic solvents (DESs) in the context of plastic-injecting molding (PIM) can be significantly improved through careful molecular design. By incorporating functional groups such as carboxylates, thiols, or phosphoryl moieties into the structures of ILs or DESs, we can achieve targeted coordination with soft or borderline metal cations. This approach allows for the fine-tuning of extraction behavior while maintaining low volatility and high thermal stability. Moreover, when coupled with biodegradable matrices such as PLA or chitosan, these carriers form fully integrated membrane systems with minimized environmental footprints. Looking ahead, research may benefit from synergistically combining bio-based carriers with natural antifouling agents. For instance, embedding DESs that contain antimicrobial organic acids or amino acids into membranes functionalized with plant-derived biopolymers could result in dual-function systems—simultaneously selective and resistant to microbial contamination. This concept, as proposed by Kandel et al., represents a forward-thinking strategy for water and wastewater treatment membranes that fulfill both operational and ecological performance metrics [48]. Table 2 provides a comparison of the most relevant carriers and their selective behavior toward different target ions. This shows the variety of options available to researchers for customizing PIM functionality for specific applications.

The future direction of carrier development for PIMs is moving toward multifunctional and stimuli-responsive extractants, including smart ILs capable of pH- or redox-controlled binding and biomimetic carriers inspired by metalloproteins. In parallel, efforts are underway to minimize carrier leaching and toxicity through covalent immobilization or encapsulation within nanoporous frameworks.

These innovations in carrier chemistry have improved the selectivity and efficiency of PIMs. They have expanded their application scope to include critical metal recovery, nutrient recycling, pharmaceutical separation, and sensing technologies, positioning PIMs as a versatile and sustainable option in modern separation science.

### 2.2. Polymer Matrix Functionalization and Its Impact on Selectivity

While the transport carrier is responsible for analyte recognition, the polymer matrix in which it is immobilized plays an equally critical role in defining the physical and chemical environment for transport. The structure and functionality of the matrix influence membrane permeability, ion–carrier interaction frequency, and even the system’s long-term stability. Thus, tuning the polymer matrix is a key strategy for enhancing the selectivity of PIMs, particularly when dealing with multicomponent mixtures or low-concentration analytes.

A major direction in matrix optimization involves introducing functional groups into the polymer backbone. The sulfonation of standard matrices, such as PVC and CTA, introduces negatively charged sulfonate (-SO_3_^−^) groups, which improve electrostatic attraction toward multivalent cations and facilitate diffusion. Studies have shown that sulfonated CTA, membranes yield significantly higher fluxes and improved separation factors for metal ions such as Pb^2+^, Cu^2+^, and Cr^3+^ compared to their non-sulfonated analogs [65].

Another functionalization pathway is carboxylation, which is performed by grafting maleic anhydride or blending with poly(acrylic acid). This modification adds carboxylic acid (-COOH) groups with strong metal-chelating properties. Carboxylated membranes have been especially effective in the selective transport of rare-earth elements and transition metals under acidic conditions. They improve coordination and membrane stability [66,67].

In addition to chemical groups, adding nanomaterials into a polymer matrix has become an important method for modifying membrane structure and selectivity. Nanofillers such as graphene oxide (GO), titanium dioxide (TiO_2_), and halloysite nanotubes increase the membrane’s internal surface area and provide additional reactive sites for ion binding. For example, GO-functionalized CTA membranes showed a twofold increase in Pb^2+^ transport efficiency, attributed to better carrier dispersion, higher porosity, and reduced fouling [68]. Moreover, the nanostructure of these additives can create complex transport pathways that limit the unwanted diffusion of non-target species, improving overall selectivity. Nanomaterials are not just passive fillers; they are increasingly being used as active transport enhancers and ion carriers in electrochemical systems. Recent advancements in membrane engineering indicate that it is possible to mimic PIM-like behavior by using dual-layer electrospun membranes that are embedded with functional nanoparticles. Electrospinning allows for precise control over fiber diameter, alignment, and surface chemistry, enabling the creation of asymmetric structures where one layer provides selective ion recognition while the other promotes directional flux. For example, GO or TiO_2_-loaded electrospun membranes can act as selective ion-transport channels in capacitive or electrochemical sensing systems. Their high conductivity and tunable surface chemistry facilitate charge transfer and selective electrostatic interactions with target analytes. This strategy closely parallels PIM design in terms of carrier-mediated transport, yet offers enhanced surface-to-volume ratios and greater adaptability for sensor miniaturization. A recent study by Kwak et al. (2025) showed that integrating metal–organic frameworks (MOFs) and 3D nanostructures into bipolar membranes significantly decreased the water dissociation overpotential to thermodynamic limits, highlighting the synergistic potential of nanoarchitectures in separation and sensing [69]. Such findings support the integration of functional nanomaterials into electrospun membranes as a pathway to develop PIM-inspired, hybrid sensing and separation systems with advanced performance metrics.

Another trend is the use of bio-based fillers in membrane production. Materials such as acetylated lignin, starch derivatives, and cellulose nanocrystals contribute functional binding groups (e.g., hydroxyl, phenolic, or amine) and improve the membrane’s mechanical integrity and sustainability. These renewable materials reduce production costs and lessen the environmental impact. For instance, CTA membranes blended with acetylated lignin and D2EHPA efficiently extracted Ni^2+^ and Co^2+^ from wastewater while maintaining over 85% performance across multiple transport cycles [70].

An often-overlooked aspect of matrix modification is its indirect influence on the carrier’s behavior. Hydrophilic modifications, such as sulfonation or nanoparticle inclusion, can increase the mobility and availability of the carrier phase. They do this by preventing clumping and improving its distribution within the membrane. Simultaneously, polar functional groups in the matrix can anchor mobile carriers, reducing leaching and prolonging the membrane’s life.

**Figure 3:**
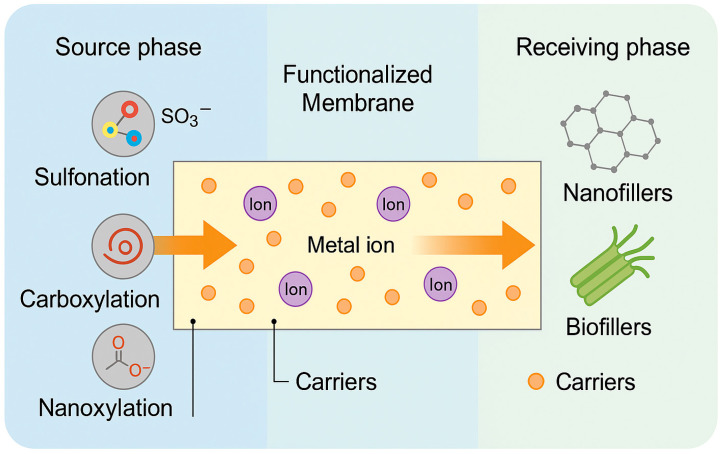
Matrix functionalization strategies and their effect on ion transport in PIMs.

These combined effects on ion–carrier interaction, membrane porosity, phase retention, and fouling resistance highlight the pivotal role of matrix design in controlling selectivity. The diverse functionalization strategies and their impact on transport behavior are conceptually illustrated in Figure 3, which summarizes how sulfonation, carboxylation, nanofiller integration, and biofiller blending shape ion migration from the source phase to the receiving phase across the membrane.

A schematic representation of polymer matrix functionalization strategies and their impact on transport selectivity in PIMs illustrates ion migration across a functionalized membrane from the source to the receiving phase.

In summary, polymer matrix functionalization provides a complementary route to carrier design for tuning PIM selectivity. Whether by modifying surface charge, incorporating diffusion enhancers, or embedding biofunctional moieties, such alterations redefine how ions interact with the carrier and the membrane phase. These strategies not only expand the use of PIMs to more complex separation problems but also match their design with the goals of sustainability, reusability, and long-term stability, core criteria for new environmental and industrial challenges.

## 3. Enhancement of Mechanical and Chemical Stability

Durability under mechanical stress and chemical exposure remains a significant challenge for polymer inclusion membranes (PIMs), especially in industrial and environmental applications. Over the last five years, we have made significant progress by using better polymers and cross-linking techniques. Membrane integrity has been reinforced while performance has been preserved or enhanced.

### 3.1. Utilization of Advanced Polymers

The choice of base polymer matrix is critical in determining the mechanical, thermal, and chemical resistance of polymer inclusion membranes (PIMs). Traditional matrices such as cellulose triacetate (CTA) and polyvinyl chloride (PVC) have shown good compatibility with various carriers. However, their use is often limited under harsh conditions due to plasticizer leaching, swelling, and degradation in acidic or organic environments [65,71,72].

To address these challenges, recent research has increasingly focused on advanced engineering polymers such as poly(vinylidene fluoride) (PVDF), poly(vinylidene fluoride-co-hexafluoropropylene) (PVDF-HFP), and polyether ether ketone (PEEK). These materials offer enhanced mechanical stability, chemical inertness and thermal resistance, enabling membrane operation in more aggressive environments without sacrificing selectivity or permeability [29,72,73].

#### 3.1.1. PVDF- and PVDF-HFP-Based PIMs

**Figure 4:**
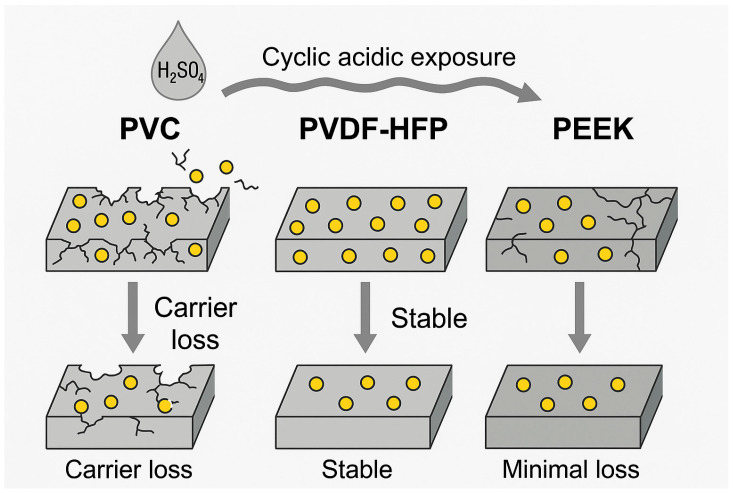
Schematic representation of membrane behavior under aggressive chemical conditions for different polymer matrices (PVC, PVD-HFP, PEEK), showing comparative carrier retention and structural degradation across operating cycles (🟡—Carrier).

PVDF-HFP has emerged as one of the most promising membrane-forming polymers due to its superior flexibility, hydrophobicity, and chemical inertness. The presence of fluorine atoms confers high resistance to oxidative and acidic attack, while the amorphous HFP segments promote elasticity and improve membrane processability [72]. Recent studies have shown that PVDF-HFP-based PIMs functionalized with D2EHPA exhibit significantly longer operational lifespans and reduced carrier loss compared to CTA- or PVC-based counterparts. Kazemi and Yaftian (2024) demonstrated that PVDF-HFP membranes functionalized with D2EHPA retained over 90% of their Bi(III) extraction efficiency across five consecutive acidic transport cycles, while CTA membranes lost structural integrity under the same conditions [29]. Moreover, PVDF-HFP exhibits lower water uptake and swelling, which contribute to dimensional stability and reduced carrier loss [28]. A visual summary of these benefits is presented in Figure 4, which compares the structural behavior of PIMs based on PVC, PVDF-HFP, and PEEK under repeated acidic exposure. The superior retention of membrane structure and carrier content in PVDF-HFP and PEEK membranes is evident.

#### 3.1.2. PEEK-Based PIMs

Polyether ether ketone (PEEK) in PIMs is not as widely studied because it is complex to process and expensive. It represents a class of ultra-durable, high-temperature polymers used extensively in aerospace and biomedical applications. Its semicrystalline structure provides exceptional tensile strength (>100 MPa), thermal resistance (up to 250 °C), and chemical resistance even to strong acids and chlorinated solvents [74]. In exploratory studies, PEEK-based PIMs have shown no visible degradation after 96 h of exposure to concentrated HNO_3_ and HCl, thereby maintaining their transport properties and structural integrity. These characteristics make PEEK suitable for long-term, high-pressure, or high-temperature separations in industrial wastewater treatment and analytical sensing [30].

#### 3.1.3. Comparative Properties and Potential Applications

High-performance polymer matrices such as PVDF-HFP and PEEK provide notable benefits compared to traditional materials like PVC and CTA. Their enhanced physicochemical properties broaden the range of environmental and industrial conditions in which PIMs can function effectively.

- Wider pH Stability: PVDF-HFP and PEEK maintain stability across a broad pH range of approximately 1 to 10, making them suitable for use in both acidic and alkaline environments.
- Enhanced Thermal Resistance: PVDF-HFP can withstand temperatures up to around 120 °C, while PEEK remains stable at temperatures exceeding 250 °C. This capability allows for applications in thermally intensive processes.
- Improved Carrier Retention: Due to their lower surface energy and dense polymeric structure, these matrices reduce carrier volatilization and leaching, thereby extending membrane lifetime and selectivity.
- Antifouling Behavior: Their hydrophobic and chemically inert surfaces minimize organic and microbial adhesion, resulting in lower fouling rates and reduced cleaning frequency.

PVDF-HFP and PEEK exhibit performance features that make them highly suitable for demanding applications, such as wastewater treatment, metal recovery, and gas separation under harsh conditions. Table 3 presents a comparative overview of the key physicochemical parameters for commonly used PIM matrix polymers, including tensile strength, thermal stability, carrier retention capacity, and operational longevity.

Additionally, these advanced matrices show excellent compatibility with electrospinning, solvent evaporation, and casting techniques, facilitating the development of nanostructured PIMs or multi-layered composite architectures [27].

### 3.2. Cross-Linking Strategies and Stability Under Harsh Conditions

Ensuring structural durability and carrier retention in polymer inclusion membranes (PIMs) is crucial, especially under demanding thermal, chemical, or mechanical stress. Cross-linking methods—whether UV/light-initiated, thermal, or chemical—have emerged as practical approaches to improve membrane strength without losing transport performance.

Controlled cross-linking creates interlocked polymer networks that limit plasticizer-induced swelling, reduce carrier leaching, and improve mechanical response. For example, the UV-initiated addition of poly(ethylene glycol) dimethacrylate (PEG-DMA) into PVDF-HFP membranes forms a semi-interpenetrating polymer network (semi-IPN) that significantly increases tensile modulus by 25%, lowers membrane swelling by over 50%, and maintains Pb(II) flux nearly unchanged after 10 reuse cycles [27,75]. Thermal cross-linking techniques, such as mild heating of PVDF-HFP with difunctional linkers have also shown notable improvements. Membranes subjected to 80 °C curing preserved over 90% ion-selective transport activity after exposure to 0.1 M HCl for 72 h, compared to a 40% drop in non-cross-linked counterparts [76]. Chemical cross-linkers, such as glutaraldehyde and epichlorohydrin, have been used with CTA and PVC matrices to resist organic solvents and surfactant-rich environments. These membranes displayed 20–40% improved longevity, extending workable lifetimes from ~5 cycles to more than 8 in industrial wastewater simulations [9].

Beyond chemical resistance and swelling suppression, the long-term performance of PIMs in aggressive environments depends on several interrelated factors: (i) polymer matrix stability, (ii) carrier–polymer compatibility, (iii) plasticizer migration behavior, and (iv) membrane microstructure. For example, exposure to strong oxidizing agents (such as ClO^−^ and H_2_O_2_), extreme acidic or alkaline pH levels, or high ionic strengths can speed up polymer degradation or cause carrier leaching in non-cross-linked systems. Cross-linking helps reduce these effects by limiting segmental mobility and forming a cohesive polymer network that can better withstand hydrolytic and oxidative stress. A detailed study on CTA-based membranes demonstrated that glutaraldehyde-induced cross-linking preserved 85% of Pb^2+^ transport capacity after 15 cycles in wastewater spiked with detergents and oils. In contrast, uncross-linked controls lost 50% of capacity after only six cycles [77].

Similarly, UV-cross-linked PVDF-HFP membranes retained their performance after prolonged exposure (120 h) to simulated acidic mine drainage (pH ≈ 2.5), maintaining stable Zn^2+^ and Cu^2+^ permeability. This was attributed to enhanced phase compatibility between ionic liquid carriers and the semi-IPN structure, which immobilized the functional phase and prevented leaching [78]. Temperature also plays a critical role. Membranes operated at ≥60 °C often exhibit accelerated plasticizer migration or phase separation. However, cross-linked membranes (particularly those thermally or UV-cured) showed limited deterioration in selectivity and structural integrity even after thermal cycling between 20 °C and 80 °C [79]. The use of cross-linking also improves resistance to shear stress in continuous flow or pressurized systems. For example, membranes reinforced with epoxy or multifunctional acrylates retained their tensile strength and functional flux after more than 50 h of convective flow at 2 bar, indicating their applicability in pilot-scale treatment modules [80]. Table 4 highlights the distinct impact of cross-linking routes on mechanical strength, swelling degree, ion flux stability, and operational lifespan across various polymer matrices and conditions.

**Figure 5:**
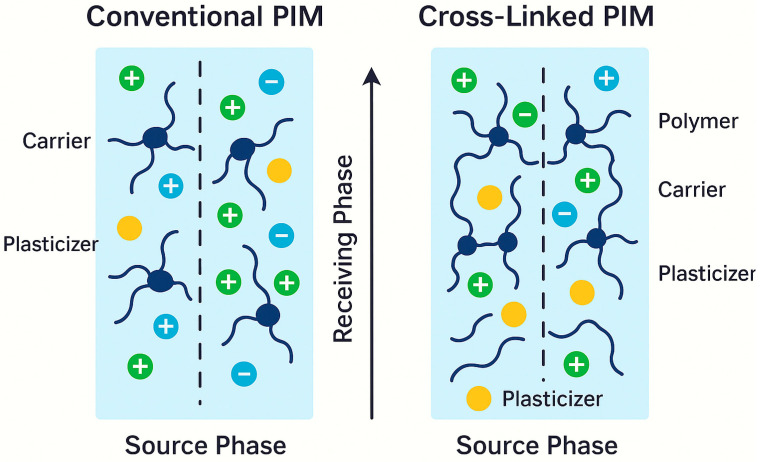
Schematic comparison of conventional vs. cross-linked PIM structures (🟡—Carrier; ⚫—Plasticizer; 🔵—Anion; 🟢—Cation).

Moreover, Figure 5 schematically illustrates the difference between the structures of conventional and cross-linked membranes, showing how networked polymer structures more effectively retain carrier molecules and reduce plasticizer mobility, thereby enhancing stability in aggressive environments.

Overall, strategic cross-linking strengthens membrane structure, minimizes plasticizer and carrier bleed, and creates a stable environment for facilitated transport—even in aggressive chemical or thermal scenarios. This enables PIMs to compete against conventional membranes, especially in industrial or environmental applications requiring stringent durability.

## 4. Environmental Applications of PIMs

Polymer inclusion membranes are increasingly used for environmental remediation, particularly for removing heavy metals and organic pollutants from water, as well as for gas separation, and carbon dioxide capture. Their tunable selectivity, combined with their structural stability, makes them highly promising for real-world applications.

### 4.1. Heavy Metal and Organic Pollutant Removal from Water

One of the most established and effective uses of polymer inclusion membranes (PIMs) is the selective removal of hazardous inorganic and organic pollutants from aqueous environments. These pollutants include transition metals (e.g., Pb^2+^, Cd^2+^, Ni^2+^, and Cu^2+^), toxic metalloids (e.g., Cr^6+^, As^5+^), and new organic contaminants such as pharmaceuticals, pesticides, and industrial dyes. PIMs provide a unique advantage due to their high ion selectivity, low carrier loss, and reusability under varying environmental conditions.

#### 4.1.1. Mechanism of Removal

**Figure 6:**
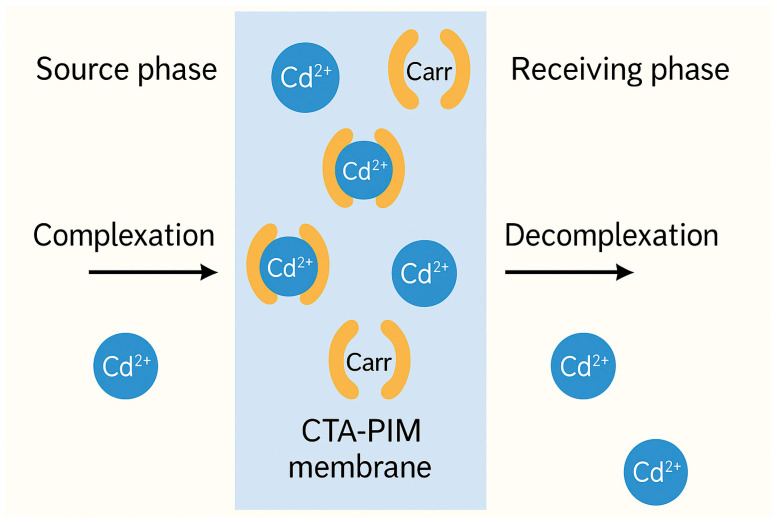
Schematic representation of Cd^2+^ transport across a CTA-based PIM from the source to receiving phase, showing complexation/decomplexation with the carrier.

PIMs operate via facilitated transport, where the embedded carrier forms a reversible complex with the target pollutant in the source phase. This complex then diffuses through the membrane and dissociates in the receiving phase, driven by a concentration gradient or pH difference. Figure 6 illustrates this process for a representative divalent cation (e.g., Cd^2+^).

#### 4.1.2. Heavy Metal Removal

Recent studies demonstrate that ionic liquid (IL)-based PIMs exhibit excellent performance in selectively removing Pb^2+^ and Cd^2+^ ions from aqueous solutions. For instance, Hernández-Fernández et al. (2023) employed a CTA-based PIM containing [Bmim][PF_6_] as an extractant, achieving greater than 90% Cd^2+^ removal within 2 h, with minimal leaching and stable operation across five cycles [27]. ILs also suppress carrier volatilization and improve membrane stability.

Biofiller-modified PIMs further enhance selectivity and membrane lifespan [36]. Kaczorowska et al. (2022) reported a bio-based CTA membrane blended with acetylated lignin and D2EHPA, which demonstrated consistent the removal of Ni^2+^ and Co^2+^ from real wastewater while maintaining its structural integrity under varying pH conditions [35].

#### 4.1.3. Organic Micropollutant Removal

PIMs have also been successfully applied to emerging contaminants, particularly pharmaceuticals. Sulfamethoxazole and diclofenac are frequently detected in surface waters, posing ecological threats. Studies using Aliquat 336-loaded PVC-based PIMs revealed rejection efficiencies of 85–95% for these drugs, depending on membrane thickness and phase pH [77]. Functionalization with sulfonic acid or carboxyl groups further enhances interactions with zwitterionic drug species. Additionally, studies have reported the successful separation of dyes using PIMs modified with cellulose nanocrystals, which offer hydrogen bonding and π-π interactions with aromatic pollutants. This approach was shown to reduce methylene blue concentration by up to 90% in simulated wastewater [30].

Polymer inclusion membranes (PIMs) provide excellent selectivity and reusability, but it is important to compare their performance with other membrane technologies. Various techniques, including nanofiltration (NF), reverse osmosis (RO), ultrafiltration (UF), and hydrogel-based membranes, have commonly been used for pollutant removal. However, each of these methods has its own specific limitations. For instance, reverse osmosis and NF provide high rejection rates for heavy metals and pharmaceuticals, but they operate under high pressure, consume more energy, and are prone to membrane fouling. Ultrafiltration membranes, including polymer-enhanced ultrafiltration (PEUF), can effectively remove specific micropollutants and macromolecules; however, they often require additives (e.g., oxidized alginate) to achieve enhanced separation and encounter challenges with selectivity and membrane clogging [78]. Hydrogel membranes are known for their swelling-controlled transport and stimulus-responsiveness, but they generally lack chemical robustness under variable pH or ionic strength conditions, and long-term reusability is often limited.

In contrast, PIMs demonstrate the following:

- High ion selectivity due to the molecular specificity of the carriers;
- Low energy demands, operating under ambient pressure and temperature;
- Minimal leaching and long-term reusability, especially when incorporating ILs or bio-based matrices;
- Tailorability for specific targets, including non-ionic pharmaceuticals, nutrients, or charged dyes;
- Greater chemical resistance, particularly with CTA, PVDF-HFP, or IL-based systems.

PIMs are particularly appealing for niche applications that require selective separation under mild conditions. These include tasks such as removing specific antibiotics, pesticides, or nutrient ions like phosphate and ammonium. Additionally, their compatibility with renewable carriers and biodegradable polymers makes them an attractive option for sustainable remediation efforts.

#### 4.1.4. Practical Benefits and Benchmarks

The principal advantages of PIM-based systems in environmental remediation include

- Exceptional selectivity toward specific target analytes, enabled by the molecular recognition capabilities of tailored carriers;
- Energy efficiency, as separation processes are typically conducted under ambient- temperature and- pressure conditions;
- Operational simplicity and renewability, with facile carrier reactivation and membrane reuse achievable through controlled pH modulation between phases;
- Enhanced sustainability, particularly when incorporating biodegradable or bio-derived fillers that reduce the environmental impact of membrane fabrication and disposal.

A performance comparison is summarized in Table 5.

### 4.2. Gas Separation and Carbon Capture

Polymer inclusion membranes (PIMs) have recently gained considerable attention in the field of gas separation, particularly for their potential application in carbon dioxide (CO_2_) capture from industrial flue gases, natural gas purification, and biogas upgrading. In contrast to traditional dense polymer membranes, which rely on solution–diffusion mechanisms, PIMs can exploit facilitated transport processes, wherein the polymer matrix incorporates selective carriers (e.g., ionic liquids, amines) that interact reversibly with CO_2_ molecules. This enables PIMs to achieve enhanced permeability and selectivity, even exceeding the classical Robeson upper bound for polymer membranes [79,80,82,83,84].

**Figure 7:**
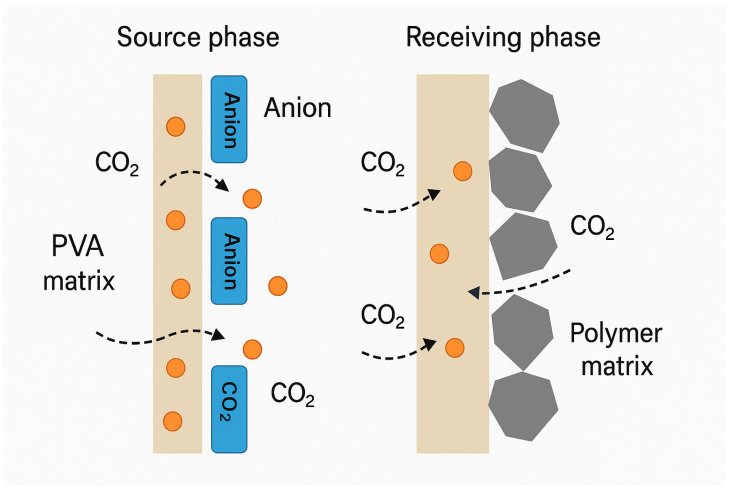
Schematic of CO_2_ transport through IL-based and GO-enhanced PIMs (🟠—CO_2_ molecule; ⬣—Graphene Oxide (GO) nanosheet or nanofiller [38].

The chemical affinity between the carrier and CO_2_ typically drives the facilitated transport mechanism in gas-separating PIMs. Ionic liquids (ILs), for instance, can form weakly bound complexes with CO_2_, promoting selective uptake on the source phase and rapid release on the permeate side. Similarly, amine-functionalized carriers react with CO_2_ via carbamate formation, facilitating directional transport. These interactions not only improve selectivity over non-reactive gases, such as N_2_ or CH_4_ but also contribute to increased diffusion fluxes due to the maintenance of concentration gradients across the membrane. A schematic representation of this process is shown in Figure 7, which compares IL-based and GO-enhanced membrane structures for the selective transport of CO_2_.

Recent studies report outstanding performance metrics for PIMs in CO_2_ separation. For example, PVA membranes incorporating aniline-based ionic liquids deposited on polysulfone supports achieved CO_2_/N_2_ selectivity values of approximately 30 and CO_2_ permeabilities approaching 300 GPU under ambient conditions [82,83,85,86]. Similarly, mixed-matrix PIMs composed of PIM-1 and graphene oxide nanosheets demonstrated CO_2_ permeabilities exceeding 6000 Barrer, coupled with CO_2_/N_2_ selectivities above 120 [82,87]. These results are primarily attributed to the high free volume of PIM-1 and the additional diffusion channels introduced by the nanofillers. Another noteworthy example is the blending of PIM-1 with poly(ethylene glycol) (PEG), which not only enhances polymer–gas interaction but also improves CO_2_/CH_4_ separation performance, reaching permeability values of around 2000 Barrer and selectivity of approximately 39 [82,88,89]. These representative data are summarized in Table 6, offering a comparative overview of selected PIM-based gas separation systems.

In addition to their promising transport properties, PIMs exhibit advantageous features in terms of stability and process adaptability. The incorporation of ILs such as [Bmim][PF_6_] or amino-functionalized ILs contributes to thermal and chemical resistance, mitigating aging and performance loss even under humid or pressurized conditions [81,89]. Moreover, the structural reinforcement provided by inorganic fillers such as graphene oxide (GO), polyhedral oligomeric silsesquioxanes (POSS), and titanium-based nanomaterials improves stability [82,90].

The manufacturing scalability of gas-separating PIMs has also been demonstrated using established thin-film fabrication techniques, such as non-solvent-induced phase separation (NIPS), spin coating, and doctor blade casting. These methods enable the formation of selective layers as thin as 1.0–10.0 µm, supported on porous polymer substrates, which provide high gas fluxes and mechanical robustness. Notably, these membranes maintain stable performance across multiple gas cycles and varying humidity conditions, supporting their potential for real-world CO_2_ removal systems [91].

In summary, gas separation and carbon capture using PIMs represent a frontier area of membrane science, combining the molecular precision of selective carriers with the process advantages of polymeric films. The integration of ionic liquids and functional nanofillers into PIM matrices provides a robust route to high-performance CO_2_ separation, offering tunable selectivity, enhanced energy efficiency, and improved environmental compatibility.

## 5. Integration of PIMs in Sensing Technologies

Polymer inclusion membranes have transcended their traditional role in separation, emerging as versatile components in optical and electrochemical sensors. By immobilizing carriers, chromogenic reagents, or electroactive elements within a polymer matrix, PIMs combine high selectivity, signal amplification, and portability, enabling robust detection platforms for environmental monitoring and analytical applications [92,93,94].

### 5.1. Optical and Electrochemical Sensors Based on PIMs

Polymer inclusion membranes (PIMs) have evolved from mere separation barriers into highly effective sensing platforms thanks to their capacity to immobilize selective carriers, chromogenic reagents, or electroactive compounds within a planar polymer matrix. This versatility enables the development of both optical sensors (optodes) and electrochemical sensors, combining selectivity, portability, and analytical precision in one compact form.

In optical PIM-based sensors, a colorimetric reagent such as a Schiff base, azo dye, or ionic liquid complex is embedded within a cellulose triacetate (CTA) or PVC membrane. These reagents undergo visually detectable color transitions–observable by the naked eye or monitored via spectrophotometry–upon interacting with specific analytes. Optical PIM-based sensors operate through a specific chemical reaction or complexation process involving an immobilized reagent (such as a dye) and an analyte (such as a metal ion). This interaction alters the electronic structure of the reagent, causing a shift in light absorption or emission bands, which is observed as a visible color change [71,95,96]. Typically, this is the result of ligand–metal complex formation, where the reagent acts as a coordinating ligand. Depending on the type of dye and the target ion, the color change may result from tautomerism, protonation/deprotonation, or electronic transitions such as metal-to-ligand charge transfer (MLCT) [92]. The polymer matrix, typically made of CTA or PVC, is essential for stabilizing the sensing system. It provides mechanical support for the reagent, prevents leaching, and allows for the controlled diffusion of the analyte to the reactive site. Furthermore, the physical and chemical properties of the membrane, such as porosity and hydrophilicity, affect both the kinetics and selectivity of the sensor’s response [92,95]. Thanks to these features, optical PIM sensors can provide a reliable signal in a short time frame (typically a few to several minutes), while maintaining reusability and reproducibility [96].

A noteworthy example, as cited by Sánchez-Ponce et al. (2021), employed 2-acetylpyridine benzoylhydrazone (2-APBH) immobilized within a PVC-PIM to selectively sense Cd^2+^. This sensor achieved a limit of detection as low as 0.02 mg/L, demonstrated a linear response up to 1.0 mg/L, and provided results within just 20 min [92]. Additionally, the sensor maintained reliable operation over multiple cycles and proved accurate when detecting Cd^2+^ in natural water and even art paint samples.

Beyond cadmium sensors, Cu^2+^, Zn^2+^, Fe^2+^, and Pb^2+^ optodes based on CTA-PIMs with chromophores like 1-(2-pyridylazo)-2-naphthol (PAN) have been reported. In one study, an optode incorporating TEHP and PAN detected metal ions within 20 min and demonstrated strong optical signal retention over a period of nine days. Techniques combining mathematical analysis methods, such as multivariate curve resolution, have further enhanced the quantitative accuracy of these sensors [92,97,98].

Electrochemical PIM sensors, on the other hand, typically involve coating an electrode—such as a screen-printed or glassy carbon electrode—with a CTA-PIM containing selective ionophores or ionic liquids. Carriers, such as calixarenes or ionophoric compounds, facilitate the specific transfer of ions, creating measurable potentiometric or amperometric responses. These systems offer remarkable detection limits in the parts-per-billion range, rapid response times (often below 30 s), and excellent stability, usually preserving functionality over 50 or more measurement cycles [99,100]. The operation of electrochemical sensors based on polymer inclusion membranes (PIMs) relies on the selective extraction and transport of target ions from the sample solution into the membrane phase. This process is facilitated by an ionophore or carrier that is embedded within the polymer matrix [101]. The selective transport of ions modifies the local electrochemical environment at the interface between the electrode and the membrane. For potentiometric sensors, this results in a change in phase boundary potential that is proportional to the logarithm of the ion activity, which is then measured as an electrical potential difference [102]. In amperometric systems, redox-active analytes are transferred into the membrane and oxidized or reduced at the electrode surface, producing a current directly related to analyte concentration [103]. The polymeric membrane provides physical separation between the sample and the electrode while allowing selective ion transport through embedded carriers. Ionic liquids or plasticizers are often incorporated into the polymeric ionic membrane (PIM) to improve membrane conductivity, enhance ion mobility, and ensure compatibility with the electrode surface. Additionally, immobilizing reactive components in a solid matrix boosts operational stability and reproducibility while reducing the leaching of active compounds, which is a common issue in traditional liquid membrane systems. This structural robustness allows the sensor to be reused for multiple measurement cycles without significant degradation in signal quality or selectivity.

**Figure 8:**
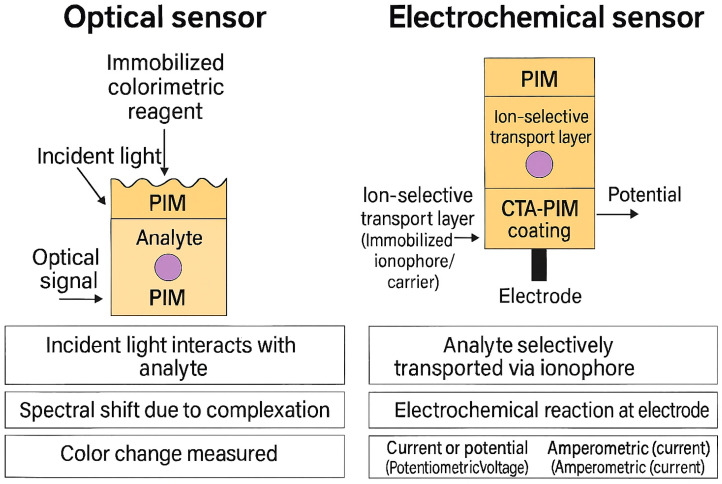
Schematic illustration of PIM-based sensing platforms: **left**–optical sensor with immobilized colorimetric reagent; **right**–electrochemical sensor with CTA-PIM coating on electrode.

What makes PIM-based sensors particularly compelling is their combination of high analytical performance, mechanical integrity, and deployment readiness—they are inexpensive, simple to fabricate, and portable. The immobilization of reactive components within a solid matrix also mitigates the leaching issues and instability often encountered in liquid membrane sensors. The working principles of both PIM-based optical and electrochemical sensors are illustrated in Figure 8.

### 5.2. Performance Matrix: Sensitivity, Selectivity, and Response Time

The analytical performance of PIM-based sensors is determined by three core parameters: sensitivity, selectivity, and response time. These factors collectively govern the practical applicability of the sensor in trace detection, environmental monitoring, and field-based diagnostics. Their values depend not only on the type of embedded reagent or carrier but also on the physical properties of the polymer matrix, the mode of signal transduction (optical or electrochemical), and the operational conditions.

Sensitivity reflects the sensor’s ability to detect minute changes in analyte concentration. In PIM-based optodes, this is typically defined by the slope of the calibration curve and the limit of detection (LOD). Sensors embedding Schiff base ligands or azo dyes in plasticized PVC or CTA matrices have achieved LODs as low as 0.01 to 0.05 mg L^−1^ for divalent metal ions such as Cd^2+^, Pb^2+^, and Fe^2+^. For example, the Cd^2+^ sensor developed by Sánchez-Ponce et al. achieved a linear response range from 0.02 to 1 mg L^−1^, with an LOD of 0.02 mg L^−1^, supporting practical deployment in environmental analysis and potable water quality control [46]. Similarly, Mancilla-Rico et al. developed a PIM-based optode capable of simultaneously detecting Cu^2+^, Zn^2+^, and Pb^2+^ using multivariate analysis of overlapping signals, enhancing both sensitivity and multidimensional selectivity [104].

Electrochemical sensors based on PIMs can achieve even lower detection thresholds–in the parts-per-billion range—owing to the synergistic effect of facilitated transport and enhanced charge transfer mechanisms. A study by Yacoob et al. reported the detection of Zn^2+^ and Cu^2+^ at sub-ppb levels using ionophore-modified PIMs on screen-printed electrodes [32].

Selectivity is another critical aspect, particularly when operating in complex or real-world matrices. The specificity of PIMs arises from the embedded carrier or ionophore, which is often designed to form highly stable complexes with the target ion. For instance, Suah et al. demonstrated that Fe^2+^-selective optodes based on CTA and PAN exhibit limited cross-reactivity toward Fe^3+^ and Zn^2+^ due to the coordination geometry and the acidic pH environment favoring Fe^2+^ complexation [105].

**Figure 9:**
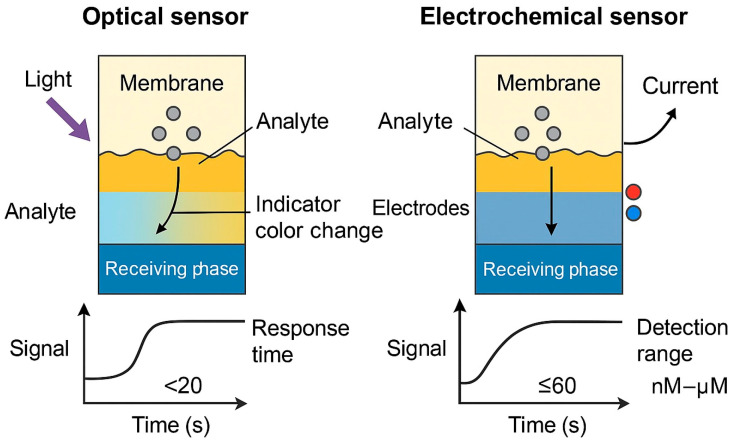
Comparative schematic of PIM-based optical vs. electrochemical sensor performance, showing signal generation modes, response time, and detection range (●: analyte molecules, ●●: electrons involved in electrochemical signal generation).

Response time is a practical determinant of how quickly the sensor can report changes in analyte concentration. Optical systems often have response times ranging from 5 to 30 min, whereas electrochemical formats can be significantly faster. PIM-coated electrodes for Cu^2+^ and Zn^2+^ reported by Yacoob et al. stabilized in under 30 s, showing excellent reproducibility [32]. Long-term operational stability, mechanical durability, and resistance to carrier leaching are also evaluated. Sensors that retain at least 90% of their signal after 10–15 measurements or a week of storage are considered highly stable [30]. Figure 9 provides a conceptual comparison of PIM-based optical and electrochemical sensing performance, while Table 7 summarizes representative metrics for selected sensor configurations.

## 6. Sustainable and Green Approaches in PIM Fabrication

As environmental concerns intensify, the PIM field is shifting toward greener and more sustainable fabrication methods. Research efforts are increasingly emphasizing the use of biodegradable polymers, renewable feedstocks, and low-toxicity solvents or plasticizers. These eco-driven strategies contribute to sustainable chemistry and circular economy principles while ensuring that membrane performance is not compromised [106].

### 6.1. Biodegradable and Renewable Polymers

In recent years, the environmental footprint of polymer inclusion membrane (PIM) production has come under growing scrutiny, leading to substantial interest in biodegradable and renewable materials. Traditional synthetic matrices such as PVC and CTA, while effective, are not inherently biodegradable and pose long-term disposal challenges. To align with circular economy principles and the Sustainable Development Goals, researchers have explored a wide range of biodegradable polymers that offer both performance and environmental benefits [107].

Poly(lactic acid) (PLA) is one of the most widely studied biopolymers for PIM matrices. Derived from renewable resources such as corn starch or sugarcane, PLA offers industrial compostability and relatively high mechanical strength. Its main drawback—brittleness—can be mitigated through blending with poly(ethylene glycol) (PEG), plasticizers, or cellulose nanofibers. PLA-based PIMs have been employed in heavy metal ion separation, demonstrating not only selectivity but also enhanced environmental safety upon disposal after use [30].

Poly(3-hydroxybutyrate) (PHB), a member of the polyhydroxyalkanoates (PHAs), is another bio-based polymer that shows promise in membrane science. PHB is synthesized via microbial fermentation and is fully biodegradable under both aerobic and anaerobic conditions [8]. Its semicrystalline structure contributes to excellent barrier properties and chemical resistance. PIMs based on PHB exhibit notable stability in aggressive aqueous environments, making them suitable for wastewater remediation.

Poly(vinyl alcohol) (PVA) is a partially biodegradable synthetic polymer often used in combination with green additives. Its water solubility and transparency make it ideal for sensor applications. Moreover, PVA exhibits excellent film-forming capability and is highly compatible with carriers such as Aliquat 336 and various ionic liquids. PVA-based PIMs have demonstrated superior mechanical durability during repeated extraction cycles, especially when cross-linked or hybridized with biofilters [28].

**Figure 10:**
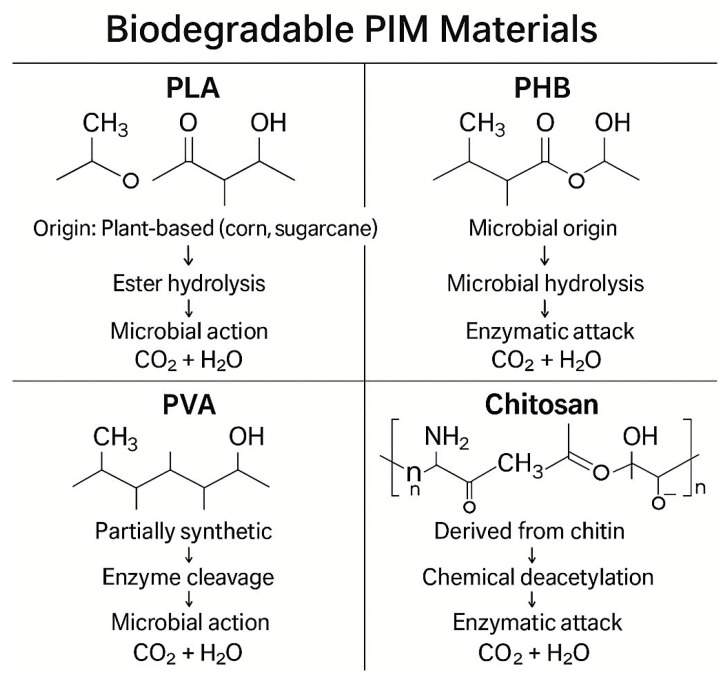
Comparative schematic of PIM-based optical vs. electrochemical sensor performance, showing signal generation modes, response time, and detection range.

Chitosan, obtained from the deacetylation of chitin, is a natural polysaccharide that is both biodegradable and biocompatible. Its amino and hydroxyl functional groups endow it with significant metal-chelating properties, allowing for the efficient removal of toxic cations like Cu^2+^, Cr^3+^, and Pb^2+^ [9]. Chitosan-PIMs can be fabricated using low-toxicity solvents such as dilute acetic acid, making the entire process more environmentally benign. Figure 10 illustrates the key structural features and degradation mechanisms associated with selected biodegradable PIM matrices, including PLA, PHB, PVA, and chitosan. The figure provides a comparative overview of molecular architecture (e.g., ester, hydroxyl, or amine groups), biodegradation pathways (such as microbial hydrolysis, enzymatic attack, or chemical cleavage), and the corresponding environmental fate of each polymer type. This structural and mechanistic analysis clarifies how each polymer enhances the performance of eco-friendly membranes and ensures safe end-of-life disposal.

A comparative summary of the properties of these biodegradable polymers, including their mechanical performance, thermal behavior, and carrier compatibility, is presented in Table 8.

### 6.2. Green Solvents and Plasticizers

As awareness grows around the ecological footprint of membrane production, the use of green solvents and plasticizers has emerged as a critical component in the sustainable fabrication of polymer inclusion membranes (PIMs). These substances not only reduce the toxicological burden associated with traditional organic chemicals but also align with the principles of green chemistry by improving energy efficiency, reducing hazardous waste, and enhancing membrane recyclability [46,106].

In conventional PIM fabrication, chlorinated solvents such as dichloromethane (DCM), chloroform, and tetrahydrofuran (THF) have been widely used to dissolve hydrophobic polymers like cellulose triacetate (CTA). However, these solvents are classified as volatile organic compounds (VOCs) and pose significant health and environmental hazards due to their high volatility, flammability, and persistence. Therefore, a paradigm shift has occurred toward utilizing green solvents, which are non-toxic, biodegradable, and derived from renewable resources. Among the most prominent are ethanol, ethyl lactate, γ-valerolactone (GVL), and ionic liquids (ILs). These solvents facilitate polymer dissolution under milder conditions, thereby reducing the risk of carrier degradation during membrane casting. For instance, ethyl lactate, derived from lactic acid and ethanol, is a low-toxicity solvent that effectively dissolves PLA and is increasingly used in the production of eco-friendly membranes [30,108].

**Figure 11:**
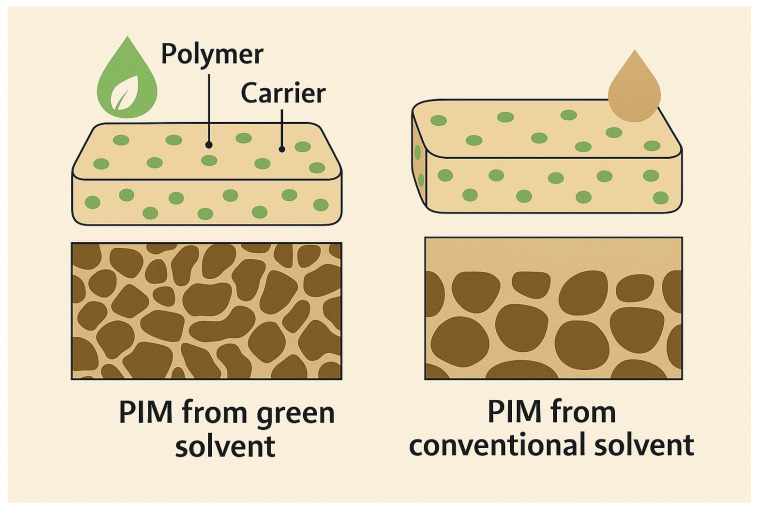
Influence of solvent type on the microstructural characteristics of polymer inclusion membranes.

Additionally, ionic liquids, particularly those based on imidazolium, pyrrolidinium, or phosphonium backbones, have gained popularity due to their negligible vapor pressure and tunable solvent properties. They are particularly effective in forming PIMs when combined with biodegradable carriers and natural polymers. As illustrated in Figure 11, membranes fabricated using green solvents often exhibit more uniform morphology, enhanced interfacial compatibility between the polymer and carrier, and greater long-term stability in aqueous media compared to membranes made using conventional VOC-based solvents [30,109,110].

Green plasticizers serve an equally essential role in sustainable PIM design by improving polymer flexibility, reducing brittleness, and maintaining carrier integrity during repeated separation cycles. Traditional plasticizers such as dioctyl phthalate (DOP) and tributyl phosphate (TBP) are effective softeners but pose significant health concerns, including endocrine disruption and bioaccumulation [111]. In contrast, plasticizers derived from natural sources such as triethyl citrate, acetyl tributyl citrate, glycerol, polyethylene glycol (PEG), and sorbitol derivatives demonstrate excellent biodegradability and minimal toxicity [62,64]. These compounds are particularly compatible with biopolymers such as PLA, PHB, PVA, and chitosan [8]. Their application in PIMs results in improved mechanical stability and reduced leaching of extractants into the surrounding environment. Table 9 provides an overview of the selected green plasticizers commonly used in PIM systems, highlighting their chemical origin, target compatibility with polymer matrices, and functional benefits. These materials support the development of safe, effective, and environmentally sound membranes that are suitable for applications in water treatment, sensing, and resource recovery.

## 7. Scalable Fabrication and Process Optimization

The ability to scale polymer inclusion membranes (PIMs) is crucial for their success in industrial settings. Along with selectivity and stability, modern fabrication techniques allow the production of membranes with a consistent structure and reproducible quality, which translates into lower processing costs and facilitates large-scale implementation.

### 7.1. Techniques: Phase Inversion, Electrospinning, and Others

The scalability of polymer inclusion membranes (PIMs) depends on how reproducible, structural integrity, and economically feasible their fabrication processes are. One popular method is the non-solvent-induced phase separation (NIPS). This technique has gained traction due to its flexibility and suitability for high-volume manufacturing. In NIPS, a polymer solution is applied to a support and then placed in a bath of non-solvent, typically water. This process starts a solvent–non-solvent exchange, which solidifies the polymer and creates a porous structure [30,35,114]. NIPS allows for precise control over porosity and membrane thickness, producing membranes that are both strong and selectively permeable [115]. NIPS works well with polymers such as cellulose triacetate (CTA), polyvinylidene fluoride (PVDF), and polyether ether ketone (PEEK), achieving porosities of up to 70% while ensuring chemical resistance.

Electrospinning is another effective method that excels at making nanofibrous PIMs. This technique uses a high voltage on a polymer solution to produce very fine fibers, typically with diameters between 100 and 500 nm. Electrospun membranes have higher surface-to-volume ratios, improved transport kinetics, and better dispersion of carriers such as ionic liquids. Electrospun PIMs are noted for their enhanced selectivity and minimal leaching, mainly when biopolymer matrices or green solvents are used [30,116]. Recent advancements such as alternating current (AC) electrospinning and needleless configurations have improved production throughput, making electrospinning increasingly viable for scale-up.

**Figure 12:**
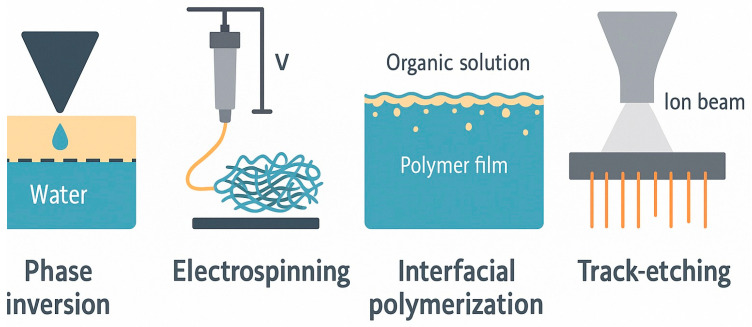
Comparison of PIM morphology using conventional and green solvents.

An emerging area is melt electrospinning writing (MEW), a technique that skips solvents and instead pushes molten polymers using electric fields to create microfibrous networks. This method provides precise control over fiber orientation and spacing, resulting in membranes with optimized mechanical pathways for selective ion transport. Although it is mostly used in research settings because of its lower output, MEW has shown exceptional potential in creating structured, custom-made PIMs for high-efficiency separations [29]. Figure 12 shows the structural differences between membranes made with NIPS, electrospinning, and MEW. It highlights their different shapes and the effects of each process.

Table 10, presented below, provides a comparative overview of these techniques in terms of operational scalability, energy demands, and membrane performance metrics.

### 7.2. Industrial Feasibility and Cost Considerations

The shift of polymer inclusion membranes (PIMs) from laboratory-scale prototypes to large-scale industrial applications depends on their cost-effectiveness, reliability during processing, and compatibility with current systems. Several techno-economic factors govern the industrial uptake of membrane technologies, including fabrication costs, operational stability under variable field conditions, reusability, and environmental compliance [117,118].

One of the primary benefits of PIM-based processes is their low energy demand, especially when compared to energy-heavy separation methods such as distillation or solvent extraction. PIMs operate efficiently under ambient pressure and temperature conditions, which removes the need for heating and vacuum systems. Energy analysis reports estimate that membrane separation processes consume nearly 90% less energy than thermal separation methods [30,119,120]. Material costs and membrane longevity are also critical. Table 11 summarizes economic indicators for typical materials used in PIM fabrication, including polymer cost per kg, number of reusability cycles, and carrier leaching resistance. Emerging biodegradable polymers like PLA and PVA provide savings due to their lower environmental disposal costs, although they may require optimization for long-term stability [29,114,120,121].

Another key factor is process integration. PIM modules can be configured in spiral-wound, hollow fiber, or cassette designs, enabling compatibility with standard water treatment and chemical recovery systems. Pilot-scale trials have shown consistent performance in removing heavy metals and recovering rare earth elements from complex mixtures. These include electroplating wastewater and acid mine drainage [122].

**Table 11:** Estimated economic parameters of materials used in scalable PIM fabrication [123].

Material	Approx. Cost ($/kg)	Reusability Cycles	Carrier Leaching	Environmental Impact
CTA	20–40	30–50	Moderate	Low
PVDF	60–90	50–80	Low	Moderate
PVA	10–25	15–30	High	Biodegradable
PLA	5–15	1020	Moderate	Biodegradable
PEEK	100–150	>100	Low	Low

## 8. Challenges and Future Perspectives

The field of polymer inclusion membranes (PIMs) has seen significant remarkable progress in recent years. There have been notable improvements in selectivity, mechanical strength, environmental compatibility, and manufacturing scalability [19,30,32,35,124]. These advancements have led to the successful application of PIMs in environmental remediation, gas separation, sensing, and analytical preconcentration. However, even with these achievements, several scientific and technological challenges still limit the complete industrial use of PIM systems [29,30,32,35,124,125]. This chapter presents at the important challenges that remain in PIM development, including material limitations, environmental risks, and engineering obstacles related to scaling up. It also looks at future strategies and research directions focused on overcoming these limitations. By identifying current bottlenecks and outlining innovative approaches—such as covalent carrier immobilization, bio-based membrane structures, hybrid nanostructures, and AI-driven design tools—this chapter sets the stage for the evolution of next-generation PIM technologies tailored for industrial and environmental resilience [19,28,30,80,126].

### 8.1. Remaining Limitations in Current PIMs

Despite the important progress in polymer inclusion membranes (PIMs), several limitations prevent their broad use in industry. One major challenge is carrier leaching, particularly in hydrophobic membranes where physical trapping does not hold up under dynamic flow or long-term exposure conditions [8,31,35,127]. This not only reduces membrane lifespan but also poses environmental risks. The cumulative leaching decreases membrane efficiency and may introduce secondary contamination into treated media. This issue is especially critical in membranes fabricated with biodegradable matrices such as polyvinyl alcohol (PVA) or polylactic acid (PLA), which are prone to degradation under acidic or basic pH conditions. Membranes relying solely on physically entrapped carriers–without covalent bonding or cross-linking–suffer from poor reusability. To tackle this issue, recent strategies have looked at the covalent grafting of extractants to polymer backbones or encapsulating carriers within nanoporous supports, such as mesoporous silica or cyclodextrin frameworks, to improve stability and retention [128,129].

At the same time, a significant technical challenge is the limited pH and temperature range that conventional PIMs can tolerate. Most PIMs, especially those based on cellulose triacetate (CTA), degrade or deform under aggressive industrial conditions involving high ionic strength, oxidative species, or thermal fluctuations. While engineering polymers such as polyether ether ketone (PEEK) and polyvinylidene fluoride (PVDF) provide better resistance, their use is restricted due to the need for harmful solvents, such as N-methyl-2-pyrrolidone (NMP) and dimethylformamide (DMF), along with complex and expensive processing [130,131]. Current research focuses on using green solvent systems—such as γ-valerolactone (GVL)–to process these polymers without losing their performance or sustainability [130,132].

A key issue in industrial settings is the long-term breakdown of membrane structure and function. Although many PIMs show short-term stability, but their durability over multiple transport cycles is still lacking. Repeated exposure to fluctuating pH levels, salinity, and mechanical stress can cause physical damage, such as microcracking, delamination, and carrier loss. Chemical degradation, including hydrolysis and oxidative breakdown of both the polymer and extractant, further contributes to performance decay. Research has found that CTA-based membranes can lose up to 30–40% of their transport efficiency after 10 to 15 cycles, particularly in solutions containing aggressive ions such as sulfates or chlorides [30,34].

Another ongoing issue is the limited compatibility of PIMs with complex water mixtures, like industrial wastewater or natural sources that have a lot of dissolved organic matter (DOM), humic acids, and microbial contaminants. These factors lead to fouling and pore blockage, reducing selectivity and flux. Researchers are investigating surface modifications, including PEGylation and fluorination, to improve hydrophilicity and antifouling properties. However, their long-term effectiveness in real-world conditions still needs to be clearly shown [133,134].

From a theoretical perspective, the understanding of ion transport in PIMs is still not complete. While empirical models, such as Fick’s law-based equations and facilitated diffusion approximations, provide basic predictive capability, they often fail to capture the complex interplay between the polymer matrix, carrier–analyte interactions, membrane hydration, and competing species. The lack of valid transport models limits rational membrane design [135]. Recent advances in analytical techniques, such as operando FTIR spectroscopy, X-ray photoelectron spectroscopy (XPS), and molecular dynamics simulations, provide promising ways to understand these mechanisms. However, their integration into membrane engineering frameworks is still in its early stages [30,31,136].

Reproducibility and scaling up are major challenges, particularly when moving from laboratory casting to pilot or industrial production. Membrane performance can differ widely, even with the same formulations. This happens due to differences in polymer batch purity, plasticizer composition, ambient humidity, and drying conditions. Such variability affects quality control and consistency. Furthermore, the lack of standardized fabrication protocols for PIMs makes the translation to industrial use harder, especially when using new techniques like electrospinning, 3D micro-extrusion, or melt electrowriting [137,138].

Regulatory gaps and environmental concerns create major obstacles to the broad use of PIM technologies. Although these technologies are advertised as green and sustainable, many PIMs still use volatile organic solvents (VOCs), synthetic extractants such as Aliquat 336 or D2EHPA, and non-biodegradable materials. These substances can raise issues related to toxicity and environmental impact, especially in areas such as drinking water, agriculture, or pharmaceutical processing. There is no consistent international regulation setting acceptable limits of extractant leaching, biodegradability, and environmental effects of PIM components. Developing next-generation PIMs will need close cooperation with regulatory agencies to meet sustainability standards and public health needs [35,139,140].

Another bottleneck is the narrow operating pH and temperature window, which restricts the use of PIM use in aggressive industrial environments [131]. Additionally, while specific polymers such as PEEK or PVDF offer superior resistance, they often require toxic solvents or costly processing routes [141]. The scalability of advanced structures, such as electrospun nanofibers and MEW-written matrices, also faces technical and economic constraints. PEEK- or PVDF-based membranes offer promising alternatives due to their chemical resilience; however, their fabrication often relies on hazardous solvents, such as NMP and DMF. Efforts to adapt green solvent systems (e.g., γ-valerolactone) to dissolve engineering polymers without degrading their performance are ongoing. Moreover, uniform membrane casting at an industrial scale, especially via electrospinning or micro-extrusion, demands precise control over solution rheology, temperature, and phase separation dynamics [131,142,143].

### 8.2. Future Reasearch Directions for PIMs

As PIM technologies evolve, the next generation of research aims to overcome current limitations by incorporating innovative, adaptive, and multifunctional features into membrane systems. One major direction involves the development of stimuli-responsive membranes that can modulate their transport properties in response to external cues such as pH, temperature, or electric fields. For example, using pH-sensitive carriers, such as benzimidazole derivatives or thermoresponsive polymers like PNIPAm can provide dynamic control over ion transport. This control could greatly benefit biomedical and environmental applications [144,145].

Another promising area is combining nanotechnology with PIM architecture. Adding metal–organic frameworks (MOFs), covalent organic frameworks (COFs), or functionalized nanoparticles (e.g., TiO_2_, ZnO, Fe_3_O_4_) into polymer matrices can improve selectivity and permeability. This approach also brings in photocatalytic or antimicrobial functions. These hybrid materials could pave the way for PIMs capable of simultaneously separating and degrading of organic micropollutants [146,147,148].

In structural engineering, new fabrication methods like additive manufacturing, including direct ink writing, melt electrowriting, as well as nanoscale patterning, are being studied to create membranes with specific porosity, different channels orientations, or layered architectures. These approaches aim to optimize both flux and selectivity while reducing resistance to mass transfer [149,150,151,152].

**Figure 13:**
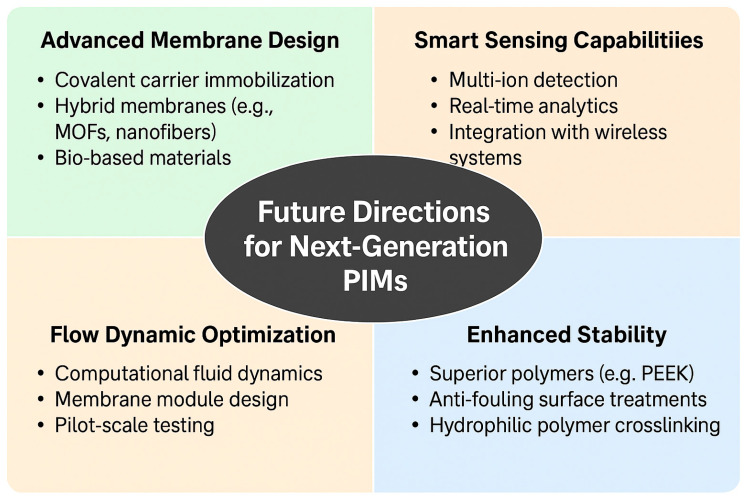
Comparative overview of future research directions.

Bioinspired designs also offer considerable potential. Mimicking biological membranes using phospholipid analogs, aquaporin-like channels, or self-assembling peptide carriers can lead to ultra-selective and energy-efficient transport. Moreover, the use of artificial ion channels or gated nanopores may introduce new paradigms for molecular recognition and selective separation [153,154]. Figure 13 illustrates the multidisciplinary innovations driving the next-generation development of PIMs, including the integration of nanotechnology, innovative materials, and bioinspired designs.

Digital tools are becoming increasingly integral to PIM research. Machine learning algorithms predict membrane performance using compositional and processing parameters. When combined with high-throughput screening and multi-objective optimization, these methods should speed up material discovery and process design [155,156].

From a sustainability perspective, next-generation PIMs will focus more on circular economy principles. This includes using waste-derived polymers, recyclable membranes, and closed-loop manufacturing systems. Combining these materials with life cycle analysis (LCA) and techno-economic analysis (TEA) will be important for measuring their environmental impact and practicality [30,157,158].

To support these improvements, multidisciplinary collaboration between chemists, material scientists, process engineers, and computational experts will be vital. Integrating new fields, such as green chemistry, artificial intelligence, and synthetic biology, could change the future of membrane science. This integration would lead to scalable, high-performance PIMs with low environmental impact [159,160,161,162,163]. Table 12 offers a comparison of emerging research directions, their goals, and related technical challenges.

## 9. Conclusions

The development of polymer inclusion membranes (PIMs) has become an important area in separation science. PIMs offer a unique mix of selectivity, structural flexibility, and environmental compatibility. In the last twenty years, there has been significant progress in material design, membrane engineering, and the range of applications. The findings presented in this manuscript, spanning fundamental chemistry, functionalization strategies, fabrication techniques, environmental applications, and prospects, highlight the multidisciplinary potential of PIM-based systems.

PIMs are highly effective in selective ion transport, particularly for heavy metals and rare-earth elements. This is thanks to their specific carrier-polymer design and improved transport methods. Developments in polymer matrix engineering, like sulfonation, carboxylation, nanofiller incorporation, and blending with biofillers, have significantly improved membrane stability, permeability, and reusability. The use of ionic liquids and smart extractants has also improved the specificity and efficiency of metal separation processes. From a sustainability standpoint, the move towards biodegradable polymers and green solvents demonstrates an increasing awareness of environmental impacts and regulatory requirements. This shift has been supported by using green plasticizers and solvent-free fabrication methods, allowing for safer membrane production without lowering performance. Scalability and industrial feasibility remain critical milestones. The successful transfer of laboratory-scale fabrication techniques, such as phase inversion, spin coating, and electrospinning, to pilot and full-scale production environments demonstrates the readiness of PIM technology. Nonetheless, standardization and quality control still pose technical challenges, particularly in achieving reproducibility and consistent membrane quality. PIMs are also gaining popularity in analytical chemistry and sensor development. Optical and electrochemical sensors based on PIM technology can quickly, selectively, and accurately detect trace analytes. This highlights their value in environmental monitoring and medical diagnostics.

Despite this progress, some limitations, such as carrier leaching, reduced lifetime in aggressive environments, and gaps in regulations, highlight the need for ongoing innovation. Chapter 8 discussed strategic research directions, including stimuli-responsive membranes, bioinspired architectures, and AI-guided membrane creation, which could help overcome current obstacles.

In conclusion, PIMs are evolving into scalable, sustainable, and multifunctional membrane solutions. The combination of green chemistry, nanotechnology, and computational tools is expected to shape the next generation of high-performance PIM systems that can tackle global challenges in clean water, resource recovery, and environmental health.

## Figures and Tables

**Table 1:** Key Milestones in PIM Development and Representative Publications.

Period	Key Developments	Representative Publications
1960s–1980s	Conceptual foundation; immobilized extractants; early SLMs	Bloch et al. [33]
1990s	Formalization of PIMs; use of CTA/PVC; Aliquat 336 and D2EHPA	Almeida et al. [9], Hakami et al. [11], Zielińska et al. [12]
2000s	Mechanistic studies; polymer-carrier optimization; initial modeling	Nghiem et al. [39], Cattrall et al. [40]
2010–2015	Broader applications in analytical/environmental science	Gherasim et al. [41], Kolev et al. [42,43]
2016–2020	ILs and DESs introduced as carriers; modeling transport of complex ions	Hernández-Fernández et al. [27], Matsumoto et al. [44], Kończyk et al. [45]
2021–2022	Green solvents and biodegradable polymers; sensor integration	Senila et al. [30], Sánchez-Ponce et al. [46]
2023–2025	Scalable production; biofillers; gas separation and industrial wastewater use	Adigun et al. [28], Kazemi and Yaftian [29], Kaczorowska et al. [35]

**Table 2:** Representative Task-Specific Carriers and Selectivity Profiles in PIMs.

Carrier Type	Example	Target Ion(s)	Observed Selectivity	Ref.
Imidazolium Ionic Liquid	[C_8_mim][NTf_2_] + Cyanex 301	Co^2+^, Ni^2+^	α(Co^2+^/Li^+^) > 30	[27,28,60,61,62,63]
Phosphonium Ionic Liquid	[P_66614_][Cl]	Ag^+^, Hg^2+^	High selectivity for soft metals	[28]
Deep Eutectic Solvent (DES)	Choline chloride + glycerol	Cd^2+^, Zn^2+^, Pb^2+^	Comparable to ILs; high for Cd^2+^, Pb^2+^	[47,48,64]
Task-Specific Macrocycle	Calix[4]arene-phosphine oxide	La^3+^, Eu^3+^	α(La^3+^/Eu^3+^) > 100	[49,50]
Crown Ether System	18-crown-6 + Aliquat 336	Na^+^, K^+^	Size-specific selectivity	[50]
Bio-based Carrier (Cyclodextrin)	Carboxymethyl-β-cyclodextrin	BPA, Phenols, Pb^2+^, Cd^2+^	Strong host–guest interaction	[51,52,53,54,55,56,57]
Task-Specific Macrocycle	Calix[4]pyrrole derivative	Ag^+^	High affinity via anion coordination	[58]
Task-Specific Macrocycle	Calixresorcinarene derivative	Pb^2+^	>90% removal, reusable	[59]

**Table 3:** Comparative properties of polymer matrices used in PIMs.

Polymer Matrix	Tensile Strength (MPa)	Max Operating Temp (°C)	Acid/Base Resistance	Carrier Leaching Tendency	Reusability	Ref.
PVC	40–50	<80	Moderate (pH 3–9)	High	Low	[65,71,72]
CTA	20–40	<90	Moderate	Moderate	Medium	[65,71,72]
PVDF-HFP	50–60	120	Excellent	Low	High	[28,29,72,73]
PEEK	>100	>250	Excellent	Very Low	Very High	[30,74]

**Table 4:** Cross-linking strategies enhancing PIM durability.

Cross-Linking Type	Polymer Base	Method	Key Benefits	Ref.
UV (PEG-DMA)	PVDF-HFP	UV + PEG-DMA	+25% modulus; −50% swelling; stable Pb(II) flux over 10 cycles	[27,75,78,79,80]
Thermal Cure	PVDF-HFP	Heat-curing at ~80 °C	>90% flux retention after 0.1 M HCl exposure over 72 h	[9,76]
Chemical (Glutaraldehyde)	CTA, PVC	Aqueous/vapor cross-linking	20–40% extended durability in solvent/surfactant conditions	[77,81]
Dual-stress reinforcement	PVDF-HFP, hybrid	Epoxy/acrylate reinforcement; flow and temp. cycling	Functional flux and tensile retention after >50 h of flow at 2 bar; stable under 20–80 °C thermal cycling	[78,79,80]

**Table 5:** Examples of PIM applications in removing metal ions and organic pollutants from water.

Application	Membrane Composition	Target Contaminants	Removal Efficiency	Operational Stability	Ref.
Heavy metal removal	CTA + [Bmim][PF_6_]	Pb^2+^, Cd^2+^	>90% in 2 h	5 reuse cycles	[27]
Biofiller-enhanced CTA	CTA + lignin + D2EHPA	Ni^2+^, Co^2+^	~88%	Maintained at pH 3–6	[28]
Pharmaceutical removal	PVC + Aliquat 336	Sulfamethoxazole, DCF	85–95%	3 reuse cycles	[30,77]
Dye removal	CNC-modified CTA	Methylene Blue	~90%	Stable after 4 cycles	[78]

**Table 6:** Performance matrix of CO_2_-facilitated transport PIMs.

Membrane System	CO_2_ Permeability (Barrer)	CO_2_ Selectivity	Notes	Ref.
IL-PVA/aniline on PSf support	≈300 GPU	CO_2_/N_2_ ≈ 30	Facilitated via IL-aniline chemistry	[82,83,86]
IL-PIM-1 + GO	~6.169	CO_2_/N_2_ ≈ 123.5	Mixed-matrix, free-volume enhanced	[82,87]
PIM-1 + PEG (2.5 wt%)	1.952	CO_2_/CH_4_ ≈ 39	Blend tailored for CH_4_ separation	[82,88,89]

**Table 7:** Representative performance metrics of selected PIM-based sensors.

Sensor Type	Target Ion(s)	LOD	Response Time	Selectivity/Reusability	Ref.
Optical (PVC-PIM)	Cd^2+^	0.02 mg/L	20 min	High/~10 uses	[104]
Optical (CTA-PIM)	Fe^2+^	~0.05 mg/L	15–25 min	Moderate/9+ days	[105]
Optical (Multianalyte)	Cu^2+^, Zn^2+^, Pb^2+^	<0.05 mg/L	15 min	With chemometric modeling	[30,32]
Electrochemical (PIM-SPCE)	Cu^2+^, Zn^2+^	ppb range	<0 s	High/>50 cycles	[32]

**Table 8:** Properties and suitability of biodegradable polymers for use in polymer inclusion membranes (PIMs).

Plasticizer	Chemical Origin	Target Polymer(s)	Biodegradability	Key Benefit in PIMs	Ref.
Triethyl citrate	Citrate ester (natural)	PLA, PHB	High	Improves flexibility, non-toxic	[8,30]
Glycerol	Biobased polyol	PVA, Chitosan	High	Enhances water retention, compatible with hydrophilic carriers	[28]
Acetyl tributyl citrate	Citrate ester (natural)	PLA, PHB	Moderate–High	Reduces brittleness, low leaching	[30,32]
Polyethylene glycol (PEG)	Synthetic, degradable	PLA, PVA	Moderate	Enhances elasticity and carrier dispersion	[28,30]
Sorbitol derivatives	Sugar alcohols	Chitosan, PVA	High	Maintains mechanical integrity and bio-compatibility	[9]

**Table 9:** Selected green plasticizers for PIM fabrication and their properties.

Polymer	Biodegradability	Mechanical Strength	Thermal Stability	Carrier Compatibility	Notable Applications	Limitations	Ref.
PLA	High (industrial composting)	Moderate	~150 °C	Moderate	Heavy metal extraction, ion-selective PIMs	Brittle, low elongation	[8,30,108]
PHB	Excellent (natural degradation)	High	~180 °C	Good	Acid/base-stable PIMs, wastewater recovery	Rigid, high crystallinity	[8,109,110]
PVA	Partial (aqueous biodegradation)	High	~200 °C	Excellent	Optical sensors, hybrid PIMs	Water-sensitive, soluble in humid air	[18]
Chitosan	Excellent (enzymatic degradation)	Low-Moderate	~120 °C	High	Metal-binding, biocompatible membranes	Acid solubility, lower durability	[112,113]

**Table 10:** Comparative analysis of scalable PIM fabrication techniques.

Technique	Fiber Size/Porosity	Throughput	Energy Demand	Solvent Required	Industrial Potential	Ref.
NIPS	5–50 µm/up to 70%	High	Moderate	Yes	Commercially established	[30,35,114,115]
Electrospinning	100–500 nm	Medium	High	Yes	Pilot to industrial	[30,116]
MEW	1–10 µm	Low–Medium	High	No	Emerging/R&D	[29]

**Table 12:** Comparative Overview of Future Research Directions in PIMs.

Research Direction	Objective	Key Challenges
Green Polymer Matrices	Develop fully biodegradable and non-toxic matrices	Limited mechanical stability and low compatibility with conventional carriers
Advanced Carrier Anchoring	Enhance carrier stability through covalent attachment or encapsulation	Synthesis complexity and limited scalability
Stimuli-Responsive PIMs	Enable selective transport triggered by pH, light, or temperature	Slow response time and material fatigue under cycling
Multiscale Modeling and Simulation	Predict transport performance under complex conditions	High computational cost and lack of experimental validation
Hybrid Membrane Structures	Combine PIMs with MOFs, COFs, or electrospun layers	Interfacial incompatibility and manufacturing difficulties

## Data Availability

Not applicable.
